# Ethical and legal issues in infectious disease research and control.

**DOI:** 10.3201/eid0707.017713

**Published:** 2001

**Authors:** C Grady, G Ramjee, J Pape, K Hofman, M Speers

**Affiliations:** National Institutes of Health, Bethesda, Maryland 20892-6705, USA.


lettuce, strawberries, and broccoli for *Salmonella*, *Shigella*, and *Escherichia coli* O157:H7. To date, 95.4% of these samples have been free of contamination. FDA has worked closely with several countries (e.g., Costa Rica, Trinidad and Tobago, Honduras, and Jamaica) to determine their needs and capacities. Examples of training and outreach include a Regional Outreach Meeting on Food Safety in Mexico City, Mexico, and one in Santiago, Chile.

To paraphrase The Future of Public Health, the 1988 Institute on Medicine report, food safety is not just what government regulators or industry quality assurance managers do. Food safety is what society does to ensure the conditions under which people can consume food that is safe, as well as wholesome and nutritious. Safe food requires the work of producers and consumers; industry and government; local, state, federal, and, increasingly, international partners.

### References

1. Mead PS, Slutsker L, Dietz V, McCaig LF, Bresee JS, Shapiro C, et al. Food-related illness and death in the United States. Emerg Infect Dis 1999;5:607-25.
2. Swaminathan B, Barrett TJ, Hunter SB, Tauxe RB, and the CDC PulseNet Task Force. PulseNet: the molecular subtyping network for foodborne bacterial disease surveillance, United States. Emerg Infect Dis 2001;7:382-9.

---

### Ethical and Legal Issues in Infectious Disease Research and Control

**Christine Grady,* Gita Ramjee,† Jean Pape‡**
**Karen Hofman,* and Majorie Speers§**
*National Institutes of Health, Bethesda, Maryland, USA; †Medical Research Council, Durban, South Africa; ‡Group Haitien d'Etude du Sarcome de Kaposi et des Infections Opportunistes, Port-au-Prince, Haiti; §Centers for Disease Control and Prevention, Atlanta, Georgia, USA

Ethical issues in international research have received an increasing amount of publicity over the past decade, as more attention is focused on global health and expanded funding is provided for research in the developing world. Much of the discussion has focused on issues that arise when researchers from countries rich in resources collaborate with researchers from poorer countries. Some of the controversies result from research on vaccines and drugs for emerging infectious diseases. This panel addressed ongoing challenges to conducting ethical research.

Christine Grady from the National Institutes of Health reviewed existing international codes and guidelines for conducting research. Ethical concerns in international research often stem from the potential for study communities in the developing world to be vulnerable to exploitation because of their social and economic circumstances. Several international codes provide guidance on the ethical conduct of clinical research including the Declaration of Helsinki, Council for International Organizations of Medical Sciences (CIOMS), International Guidelines for Biomedical Research, and the UNAIDS Guidance Document on Ethical Considerations in HIV Vaccine Research. However, these codes are recommendations, not legal imperatives. In addition, they do not address how disagreements might be resolved. Dr. Grady mentioned some current ethical dilemmas, including determination of treatment provided to participants during the course of a clinical trial, the obtaining of informed consent, obligations of researchers to the study community, and investigators' responsiveness to local health needs. Although there are no easy answers, she suggested that basic ethical principles should be applied globally, with local interpretation and implementation.

Gita Ramjee from the South African Medical Research Council discussed ethical problems and solutions used by South African researchers conducting a phase-III trial of a vaginal microbicide to prevent HIV infection. Researchers realized that participants did not understand the details of the study. Investigators then modified the process to include role playing and to allow time for trial participants to consult with peers. Researchers also added a knowledge testing component to the trial. These changes suggest that informed consent is not a one-time event but is instead an ongoing process.

The need to minimize the risk of participants' contracting HIV during the study necessitated an intensive counseling component. Participants were informed about local HIV counseling services, and sex workers were encouraged to set standard prices and to refuse clients who would not use condoms. This study shows how practical solutions can be used to reduce risks associated with research.

Finally, Jean Pape from Les Centres GHESKIO in Port-au-Prince, Haiti, highlighted the major challenges to conducting international research, which center on obtaining ethical review or institutional review board clearance. See page 547 for a more detailed article concerning this discussion.

Address for correspondence: Karen Hofman, Fogarty International Health Center, National Institutes of Health, 16 Center Drive, Building 16, Bethesda, MD 20892-6705, USA; fax: 301-594-1211; e-mail: hofmank@mail.nih.gov
